# STAT3 in Breast Cancer Onset and Progression: A Matter of Time and Context

**DOI:** 10.3390/ijms19092818

**Published:** 2018-09-18

**Authors:** Ilenia Segatto, Gustavo Baldassarre, Barbara Belletti

**Affiliations:** Unit of Molecular Oncology, Department of Advanced Cancer Research and Diagnostics, Centro di Riferimento Oncologico di Aviano (CRO), IRCCS, National Cancer Institute, 33081 Aviano, Italy; isegatto@cro.it (I.S.); gbaldassarre@cro.it (G.B.)

**Keywords:** STAT3, breast cancer, mammary epithelial cells, inflammation, dormancy, mutation, local recurrence, metastasis

## Abstract

Signal transducer and activator of transcription 3 (STAT3) is responsible for mediating the transcriptional programs downstream of several cytokine, growth factor, and oncogenic stimuli. Its expression and activity are consistently linked to cellular transformation, as well as tumor initiation and progression. Due to this central role, STAT3 is widely considered a good target for anti-cancer therapy; however, the success of these approaches has been, so far, very limited. Notably, on one side, STAT3 is aberrantly active in many breast cancers, on the other, at the physiological level, it is the main mediator of epithelial cell death during post-lactation mammary-gland involution, thus strongly suggesting that its biological functions are highly context-specific. One of the most peculiar features of STAT3 is that it can act both in cell-autonomous and non-cell-autonomous manners, simultaneously modulating the phenotypes of the tumor cells and their microenvironment. Here, we focus on the role of STAT3 in breast cancer progression, discussing the potential contrasting roles of STAT3 activation in the establishment of locally recurrent and distant metastatic disease. Based on the most recent literature, depending on the tumor cell type, the local microenvironment status, and the stage of the disease, either STAT3 activation or inactivation can support disease progression. Accordingly, cancer cells dynamically exploit STAT3 activity to carry out transcriptional programs somehow contrasting and complementary, such as supporting survival and growth, dormancy and awakening, stem cell-like features, and inflammation, immune response, and immune evasion. As a consequence, to achieve clinical efficacy, the conception and testing of anti-STAT3 targeted therapies will need a very careful evaluation of these opposing roles and of the most appropriate tumor context, disease stage and patient population to treat.

## 1. Introduction

Signal transducer and activator of transcription 3 (STAT3) belongs to the family of signal transducers and transcription factors (STATs). Among all STAT family members, STAT3 is most often correlated with tumorigenesis, as it represents a hub where virtually any signaling pathway triggered by cytokines, growth factors, and other oncogenes may converge [1,2,3]. Consistently, constitutive activation of STAT3 was demonstrated in a wide variety of human tumors, including hematological malignancies, as well as solid tumors, such as head and neck, lung, gastric, hepatocellular, colorectal, prostate, and breast cancers [1,2,3].

Aberrant STAT3 signaling was experimentally linked to both tumor initiation and progression by inducing a wide range of intracellular processes, such as inhibiting apoptosis or inducing cell proliferation, angiogenesis, invasion, and metabolism changes, as well as extracellular ones, such as extracellular matrix (ECM) remodeling, angiogenesis, and immunity [1,4]. 

There are many ways via which aberrant STAT3 activation can be achieved in human solid cancer, but it is peculiar to note that, however obtained, it is not due to activating mutations on the *STAT3* gene. This observation suggests that STAT3 activation may represent an advantage but also, in some cases, a limit for a cancer cell; for this reason, cancer evolution privileged other ways to achieve a more tunable STAT3 activation. 

Aberrant STAT3 activation often arises in a paracrine manner from oversupply of growth factors (epidermal growth factor, EGF, hepatocyte growth factor, HGF, transforming growth factor α, TGFα, etc.) and/or inflammatory cytokines (interleukin 6, IL-6, family members, etc.) within the tumor microenvironment. Alternatively, the activation of oncogenes, inactivation of tumor-suppressor genes, and other genetic events in the neoplastic cells can directly trigger STAT3 activation as part of an autocrine pathway [1,2,3,4].

At the mechanistic level, after the binding of growth factors or cytokines to their cognate receptors on the cell surface, STAT3 is phosphorylated on the Tyr-705 residue at its C-terminus. Since cytokine receptors do not have intrinsic tyrosine kinase activity, their engagement leads to activation of receptor-associated tyrosine kinases, most prominently the janus kinase, JAK family of kinases, leading to the recruitment and phosphorylation of cytosolic STAT3. Phosphorylation of Tyr-705 results in Src homology domain 2 (SH2)-mediated, head-to-tail STAT3 dimerization and translocation to the nucleus. In the nucleus, STAT3 binds to specific STAT-responsive elements in target gene promoters, thereby inducing the transcription of those genes essential for its physiological functions [3]. STAT3 regulates the transcription of a broad panel of genes, with even contrasting functions. Some of them are involved in apoptosis, such as B-cell lymphoma, Bcl-2, Bcl-xl, myeloid cell leukemia-1, Mcl-1, and survivin, and others in cell-cycle progression, such as cyclin D1, as well as some in the epithelial–mesenchimal transition, such as Twist1 and vimentin [4,5]. Notably, many STAT3 downstream target genes encode for cytokines and growth factors, whose receptors signal through STAT3 itself, thereby providing a feed-forward loop for autocrine and paracrine STAT3 activation [6]. Recent works also discovered that several G-protein-coupled receptors (GPCRs) and Toll-like receptors (TLRs), such as TLR9 and TLR4, can activate STAT3 pathway that, in turn, upregulating the expression of certain TLRs in transformed cells, promotes tumor progression [7,8,9]. 

In this work, however, we will only briefly describe details of STAT3 modulation and functions in normal and neoplastic cells or its crucial and pleiotropic roles in the tumor microenvironment, since these topics are very well and exhaustively reviewed by very recent works [1,10]. On the other hand, we will discuss in more detail the results from recent publications that highlighted a new and even more contradictory role of STAT3 during breast cancer progression, in local recurrences and distant metastases [11,12,13].

## 2. STAT3 in Anti-Cancer Strategies

As STAT3 is widely considered a signaling molecule with oncogenic properties, it is also considered a good target for anti-cancer therapy. Substantial efforts are employed to discover novel STAT3 inhibitors that can be applied in the clinic. Since the STAT3 signaling pathway can also be blocked by targeting its upstream activators, such as IL6 and JAKs, several attempts were also made by approaching this possibility, and a large number of STAT3 inhibitors were reported, with many different mechanisms of action. However, after several years of preclinical evaluation of these inhibitors, a limited number of clinical trials are currently in progress (see Table 1) [14,15]. Non-peptide SH2-domain inhibitors were identified and exploited to inhibit the growth of cells and/or tumors with elevated levels of activated STAT3 [16]. Of these, OPB-31121, OPB-51602, and C188-9 were all evaluated in early-phase clinical trials [17,18]. An alternative method involved competitive inhibition of the interactions between STAT3 and promoter elements in target genes. For example, a 15-bp double-stranded decoy oligonucleotide, targeting the STAT3 response element in the Finkel-Biskis-Jinkins murine osteogenic sarcoma, FOS promoter, was shown to competitively inhibit STAT3 binding to DNA and to suppress tumor growth in preclinical models of different types of solid cancers [19,20]. Another approach was the use of antisense oligonucleotides, such as AZD9150, which showed promising preliminary evidence of efficacy in early-phase clinical trials and will need further evaluation [21]. The observation that most of the drugs blocking STAT3 did not fulfill the clinical trial expectations strongly supports the fact that impairing STAT3 is not an easy or linear strategy and that much more work is needed to better identify the ideal patients and/or setting for the treatment. 

Very recent data, however, reported a positive outcome from the use of silibilin, a molecule that impairs STAT3 activation via direct interaction with STAT3 [22], in cancer patients with brain metastases expressing high levels of STAT3 activation in reactive astrocytes surrounding the cerebral lesion [13]. These promising findings further strengthen the notion that very specific conditions need to be assessed and taken into account to obtain a successful anti-STAT3 therapy.

## 3. STAT3 in Normal Mammary Gland and Development

Although many studies convincingly demonstrated that activation of STAT3 is a critical event in the transformation of established breast cancer cell lines in vitro, the biological, as well clinical, significance of STAT3 activation in human mammary tumorigenesis is less clear [23,24,25,26,27]. One of the distinctive features of STAT3 is its ability to elicit different and sometimes contrasting effects under different conditions. In particular, STAT3 activities were shown to be either pro-oncogenic or tumor-suppressive according to the tumor etiology/mutational landscape, suggesting that the molecular bases underlining its functions are still incompletely understood.

In line with these observations and in sharp contrast with the putative oncogenic role of STAT3 in breast cancer, it is intriguing to note that one of the first functions ascribed to STAT3 was the induction of cell death during mammary-gland involution. Thus, in the physiological setting of the post-lactation regression of the mammary tissue, the recruitment and activation of STAT3 orchestrates a complicated and finely regulated series of events, eventually leading to involution [10,28].

In this setting, STAT3 activation in the cell membrane of mammary alveolar cells is critically regulated by the sequential action of two key cytokines, leukemia inhibitory factor (LIF) and oncostatin M (OSM). LIF acts during the first, reversible phase of involution, in which cell death goes through a lysosomal-mediated mechanism and not through apoptosis. Then, OSM leads the second phase of involution, characterized by dramatic remodeling of the mammary-gland architecture and massive extracellular-matrix degradation, which eventually causes the detachment of cells from the basement membrane and their consequent apoptosis [25,29].

## 4. STAT3 in Surgery-Induced Inflammation and Breast Cancer Local Recurrence

The involvement of STAT3 signaling is also well established in the inflammatory setting, via both cell-autonomous and non-autonomous mechanisms, by orchestrating stromal rearrangements and local, as well as systemic, immune response. In this line, STAT3 recently emerged as a key player in the development and pathogenesis of psoriasis and psoriatic-like inflammatory conditions [30]. 

Recently, we investigated the consequences of inflammation induced by surgery on breast cancer cells residually left behind after primary tumor removal [11]. It is known that surgery elicits inflammatory responses that can modify the growth kinetics of breast cancer micro metastasis [31,32] and clinical, epidemiological, and molecular studies support a strong association between inflammation and cancer [33,34]. We hypothesized that the molecular events associated with the surgery-induced inflammation and the consequent wound-healing process could provide a sort of “awakening signal” for locally disseminated residual breast cancer cells. In keeping with these hypotheses, we searched for novel and specific peri-surgical treatments, aimed at killing residual tumor cells by affecting their crosstalk with the post-surgical microenvironment [11,35,36]. One signaling pathway that we found to be strongly induced in breast cancer cells when cultured in the presence of surgical essudates (wound fluids (WF) drained from breast of patients for 24 hours after removal of primary tumor) was the STAT3 pathway [11]. We discovered that post-surgical WF contained factors that induced the enrichment of breast cancer cells with stem-like and tumor-initiating properties, and these phenotypes were specifically mediated by the activation of STAT3, but not by other STAT family members. We, thus, hypothesized and tested whether inhibiting STAT3 activity in the context of surgery-induced inflammation could result in efficient targeting of these residual breast cancer cells with stem-like phenotypes. Our findings led to the conclusion that timely acting on this critical pathway was necessary for tumor (re)initiation, i.e., administering STAT3 inhibitors just as peri-surgical schedule (three administrations: at day −1, day of surgery, day +1), whereby we were able to efficiently suppress the occurrence of breast cancer local relapse [11].

## 5. STAT3 in Breast Cancer Distant Metastases

If our findings pointed to the activation of STAT3 as an “awakening signal” for locally disseminated residual breast cancer cells in the time window of post-surgical inflammation, recent data from Yates et al. in metastatic breast cancer point, however, to a further different direction [12]. By studying the distribution of driver mutations in breast cancer metastases matched with their primary lesions, they uncovered novel and intriguing insights into the genomic evolution of these relapsed clones. Firstly, while synchronous metastases (mainly lymph nodal) were typically very similar to the primary breast tumor, metachronous distant metastases had typically one or two additional driver mutations that were specific to the metastasis sample, suggesting that growth of the metastatic clone in its new niche is favored by further genomic evolution [12]. Then, while mutations in genes frequently mutated in breast cancer, such as *TP53*, *PIK3CA*, and *GATA3*, when present, were typically found in both the primary and the recurrence samples, other mutations in less frequent cancer genes were unique to the recurrence. Among them, a number of alterations were identified on the JAK2/STAT3 pathway [12]. However, and unexpectedly, the identified variants included protein-truncating mutations, frameshift indels, and essential splice site mutations, indicating that JAK2 and STAT3 functions were lost upon mutation, thus suggesting they were operating as tumor suppressor genes in that setting. It is to note that inactivating mutations in the JAK2/STAT3 pathway were never identified in previous exome studies of primary breast cancer, despite quite a number of large and deep analyses being carried out so far. The new and, in some aspects, revolutionary information that emerges is that, in some patients with breast cancer, inactivation of JAK/STAT3 signaling can, in some way, contribute to disease progression and metastasis. A possible explanation for this apparent paradox is that JAK/STAT pathway inactivation could help these advanced tumors evade the native immune response mounted against them. In support of this possibility, loss-of-function mutations concurrent with deletion of the wild-type allele in *JAK2* were recently identified as a mechanism of resistance to checkpoint inhibitor immunotherapies in melanoma patients [37]. Another intriguing possibility is that loss of STAT3 signaling is necessary for disseminated breast cancer cells to exit from a dormant state, as recently suggested by Giaccia’s group [38]. In this study, the authors investigated the mechanisms underlying the ability of breast cancer cells to disseminate to the bone marrow and remain in a dormant state for years before eventually emerging as a clinically detectable bone metastasis. By the use of in vitro and in vivo approaches, they provide evidence that, under hypoxic conditions in the osteoblast niche, LIF receptor/STAT3 signaling confers a dormancy phenotype to disseminated breast cancer cells. Loss of this pathway activation represented a critical step in downregulating dormancy-, quiescence-, and cancer-stem-cell-associated genes, and eventually, in allowing the outgrowth of indolent tumor cells disseminated to the marrow [38]. Notably, STAT3 was already identified as a dormancy-associated gene in estrogen receptor, ER-positive breast cancer cells [39]. However, the study proves this true for strongly hypoxic sites, such as the bone marrow, but the same mechanism will not necessarily work in the same way in other metastatic niches, such as the lung or the brain. Indeed, a different situation was recently captured in the brain, where brain metastatic cells orchestrate activation of STAT3 pathway in surrounding reactive astrocytes, in order to maintain a pro-metastatic program and manipulate the host’s immune response [13]. 

## 6. Conclusions and Future Perspectives

Altogether, the most recent literature on STAT3 signaling in breast cancer quite clearly indicates that the need for activated STAT3 seems not to be an absolute dogma, but rather a context- and time-dependent dynamic event. 

**Figure 1:**
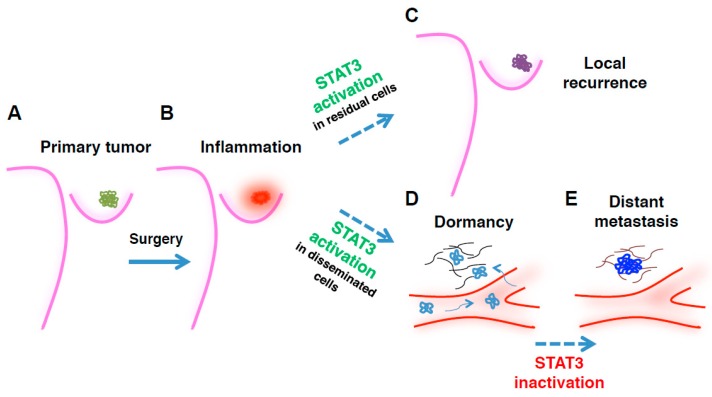
Time- and context-dependent requirement for signal transducer and activator of transcription 3 (STAT3) in breast cancer (BC) epithelial cells during disease progression. Picture depicts the proposed working model for how, where, and when STAT3 (in)activation is required by transformed mammary epithelial cells, to promote growth of primary tumor, locoregional recurrence, and/or distant metastasis. (**A**) Primary breast cancer lesions are often characterized by high levels of STAT3 activation; (**B**) Following surgical removal of the tumor mass, a surgery-induced inflammatory environment sustains, via massive local secretion of inflammatory cytokines and growth factors, the survival and re-growth of residual BC cells, at least in part via STAT3 signaling (**C**). However, in distantly disseminating BC cells, STAT3 activation can contribute to the establishment of a dormant phenotype that supports the metastatic cell survival in hostile and challenging environments (**D**). Under the pressure of cancer evolution, sub-clones carrying mutations inactivating the JAK2/STAT3 pathway emerge, overgrowing the dormant population and giving rise to frank metastases (**E**). The picture does not take into account the very relevant, and possibly contrasting, roles played by STAT3 in non-transformed cells, such as stromal cells, immune cells, and astrocytes.

A sizable number of studies ascribed a prominent role in malignant initiation and progression to activated STAT3; in breast cancer, STAT3 was found to be hyper-activated in >40% of primary tumors [40]. However, recent data also demonstrate that inactivation of STAT3 signaling can, under some circumstances, contribute to disease progression and metastasis [12,38]. Our study of the post-surgical setting shows that STAT3 signaling mediates survival and tumor (re)initiating properties in residual and locally disseminated breast cancer cells (Figure 1). However, it is believed that tumor cells have often already disseminated at distant sites at the time of diagnosis. Through mechanisms only partially known, these cells enter a dormancy state that renders them undetectable and refractory to most anti-cancer therapies, then eventually awake from dormancy and give rise to frank metastases (Figure 1) [41,42]. Thus, progressing from early to late stages of tumor dissemination, breast cancer cells can transition from a dormant to an invasive phenotype. The status and significance of STAT3 signaling can vary enormously with regards to these stages, to the microenvironment, and to the equilibrium between stimuli of growth vs. survival, and dormancy vs. awakening, needed by the cancer cell to survive in each specific moment of the disease (Figure 1) [25,43].

The presence of mutations in genes of the STAT3 pathway could have been underestimated so far, particularly in breast cancer patients at high risk of developing metastasis. Although it is possible that these mutations occur de novo at the metastatic site, their search at sub-clonal level should be pursued with most advanced and deep sequencing approaches, to evaluate whether they correlate with metastatic progression and worse prognosis.

Clarifying, at the mechanistic level, the contribution of STAT3 in tumor onset, dissemination, dormancy, and invasion, particularly in luminal breast cancer, will be very important to assess if and how STAT3 may have a future in the clinical setting. Lack of therapeutic approaches targeting disseminated dormant cells constitutes a major obstacle to the successful treatment of breast cancer patients. Thus, improving the knowledge of the mechanisms that influence dormancy/awakening switch coupled with the identification of new driver mutations that could influence metastasis occurrence certainly represents an important step for offering new effective treatment strategies to breast cancer patients at high risk of developing metastasis.

Putting together literature data, we can hypothesize that, while inhibition of STAT3 at the time and site of surgery is critical to reduce survival and growth of residual cells, its long-term impairment in cells that already reached distant sites could lead to the awakening of these dormant cells, eventually leading to formation of metastases. This could be particularly true in ER-positive breast cancer, which, differently from other subtypes, displays a propensity for very late metastatic dissemination, and where *STAT3* was identified as a dormancy-associated gene [39].

The use of appropriate preclinical models will be necessary to establish the different roles of STAT3 during disease progression in time and space, discerning when it is acting as an oncogene, as, for instance, observed in residual tumor cells in the peri-surgical microenvironment and in reactive astrocytes surrounding the brain metastases, or as a tumor suppressor, as recently observed in distantly disseminated breast cancer metastatic cells. 

STAT3 is considered a promising target in the field of cancer therapy but, until now, with limited success. For its inhibition to result in a convincing therapeutic approach, it will be critical in the future to identify the most ideal temporal treatment window and the most vulnerable (tumor) cell population to target.

## Figures and Tables

**Table 1:** Signal transducer and activator of transcription 3 (STAT3) inhibitors currently in clinical trials.

Inhibitor	Indication	Study Phase	Status	NCT Identifier
AZD9150 IONIS-STAT3Rx (STAT3 antisense oligonucleotide)	NSCLC, advanced solid tumors	I/II	Recruiting	*NCT03421353*
	Advanced pancreatic cancer, NSCLC, and CRC	II	Recruiting	*NCT02983578*
	Advanced/metastatic hepatocellular cancer	I	Completed	*NCT01839604*
	DLBCL	I	Recruiting	*NCT02549651*
	Advanced solid tumors, metastatic HNSCC	I/II	Recruiting	*NCT02499328*
	Advanced tumors, DLBCL, lymphoma	I/II	Completed	*NCT01563302*
OPB-31121 (STAT3 SH2 domain)	Advanced cancer, solid tumors	I	Completed	*NCT00955812*
	Advanced solid tumors	I	Unknown	*NCT00657176*
	Hepatocellular carcinoma	I/II	Completed	*NCT01406574*
OPB-51602 (STAT3 SH2 domain)	Advanced tumors	I	Completed	*NCT01423903*
	Multiple myeloma, NHL, AML, ALL, and CML	I	Completed	*NCT01344876*
	Advanced solid tumors	I	Completed	*NCT01184807*
OPB-111077 (STAT3 phosphorylation)	Advanced tumors	I	Completed	*NCT01711034*
Napabucasin DSP-0337 (STAT3 SH2 domain)	Metastatic pancreatic adenocarcinoma	III	Recruiting	*NCT02993731*
	Metastatic CRC	II	Not yet recruiting	*NCT03647839*
	Advanced solid tumors	I	Not yet recruiting	*NCT03416816*
STAT3 DECOY (STAT3 response element)	HNSCC	Early I	Completed	*NCT00696176*
TTI-101 (STAT3 SH2 domain)	Advanced tumors	I	Recruiting	*NCT03195699*

ALL, acute lymphoblastic leukemia; AML, acute myeloid leukemia; CML, chronic myeloid leukemia; CRC, colorectal cancer; DLBCL, diffuse large B-cell lymphoma; HNSCC, head and neck squamous cell carcinoma; NHL, non-Hodgkin lymphoma; NSCLC, non-small-cell lung cancer; SH2, Src homology domain 2; STAT3, signal transducer and activator of transcription 3.
